# Sinulariolide Induced Hepatocellular Carcinoma Apoptosis through Activation of Mitochondrial-Related Apoptotic and PERK/eIF2α/ATF4/CHOP Pathway

**DOI:** 10.3390/molecules180910146

**Published:** 2013-08-22

**Authors:** Yi-Jen Chen, Jui-Hsin Su, Chia-Yu Tsao, Chun-Tzu Hung, Hsiang-Hao Chao, Jen-Jie Lin, Ming-Hui Liao, Zih-Yan Yang, Han Hisang Huang, Feng-Jen Tsai, Shun-Hsiang Weng, Yu-Jen Wu

**Affiliations:** 1 Department of Physical Medicine and Rehabilitation, Kaohsiung Medical University Hospital, Kaohsiung 80761, Taiwan; E-Mail: chernkmu@gmail.com; 2 National Museum of Marine Biology and Aquarium, Pingtung 94450, Taiwan; E-Mail: x2219@nmmba.gov.tw; 3 Graduate Institute of Animal Vaccine Technology, National Pingtung University of Science and Technology, Pingtung 91202, Taiwan; E-Mail: d00086dog@yahoo.com.tw; 4 Department of Ophthalmology, Yuan’s General Hospital, Kaohsiung 80249, Taiwan; E-Mail: hygia1109@gmail.com; 5 English Division of the Second Faculty of Medicine, Medical University of Warsaw, Warsaw 02091, Poland; E-Mail: chaohsianghao@gmail.com; 6 Graduate Institute of Veterinary Medicine, National Pingtung University of Science and Technology, Pingtung 91202, Taiwan; E-Mails: q87634@yahoo.com.tw (J.-J.L.); mhliao@mail.npust.edu.tw (M.-H.L.); 7 Graduate Institute of Food Science, National Pingtung University of Science and Technology, Pingtung 91202, Taiwan; E-Mail: gini0307@yahoo.com.tw; 8 Department of Beauty Science, Meiho University, Pingtung 91202, Taiwan; E-Mails: hhuang.adsl@msa.hinet.net (H.H.H.); x00002036@meiho.edu.tw (F.-J.T.); 9 Department of Hospitality Management, Meiho University, Pingtung 91202, Taiwan; E-Mail: x00009520@meiho.edu.tw

**Keywords:** hepatocellular carcinoma, sinulariolide, mitochondrial, PERK/eIF2α/ATF4/CHOP, apoptosis

## Abstract

Sinulariolide, an active compound isolated from the cultured soft coral *Sinularia flexibilis*, has potent anti-microbial and anti-tumorigenesis effects towards melanoma and bladder cancer cells. In this study, we investigated the effects of sinulariolide on hepatocellular carcinoma (HCC) cell growth and protein expression. Sinulariolide suppressed the proliferation and colony formation of HCC HA22T cells in a dose-dependent manner and induced both early and late apoptosis according to flow cytometry, Annexin V/PI stain and TUNEL/DAPI stain analyses. A mechanistic analysis demonstrated that sinulariolide-induced apoptosis was activated through a mitochondria-related pathway, showing up-regulation of Bax, Bad and AIF, and down- regulation of Bcl-2, Bcl-xL, MCl-1 and p-Bad. Sinulariolide treatment led to loss of the mitochondrial membrane potential, release of mitochondrial cytochrome c to the cytosol, and activation of both caspase-9 and caspase-3. Sinulariolide-induced apoptosis was significantly blocked by the caspase inhibitors Z-VAD-FMK and Z-DEVD-FMK. The increased expression of cleaved PARP also suggested that caspase-independent apoptotic pathway was involved. In the western blotting; the elevation of ER chaperones GRP78; GRP94; and CALR; as well as up-regulations of PERK/eIF2α/ATF4/CHOP; and diminished cell death with pre-treatment of eIF2α phosphatase inhibitor; salubrinal; implicated the involvement of ER stress-mediated PERK/eIF2α/ATF4/CHOP apoptotic pathway following sinulariolide treatment in hepatoma cells. The current study suggested sinulariolide-induced hepatoma cell cytotoxicity involved multiple apoptotic signal pathways. This may implicate that sinulariolide is a potential compound for the treatment of hepatocellular carcinoma.

## 1. Introduction

Hepatocellular carcinoma (HCC), which has been reported as the most common primary malignant liver tumor, and is the third leading cause of cancer death worldwide, has received much public health attention [1]. HCC presents as an aggressive tumor type with a median survival following diagnosis of approximately 6 to 20 months [2]. Standard treatment options for HCC include surgical resection, liver transplantation, transcatheter arterial chemoembolization (TACE), percutaneous ethanol injection (PEI), radiofrequency ablation (RFA), and targeted therapy, depending on cancer stages and suitable candidates [3]. Surgical eligibility and side effects of chemotherapy agents remain as major concerns of these aggressive treatment options [4]. Therefore, agents with anti-cancer potentials and fewer side effects are more clinically preferable for cancer treatment.

The induction of apoptosis critically benefits cancer therapy development [5]. The apoptotic processes can be triggered either via extrinsic pathways by the plasma membrane or intrinsic pathways which are within cells [6,7]. Recent studies have shown that intrinsic pathways are initiated by the associations between stress and the endoplasmic reticulum (ER) or mitochondria [8]. ER is responsible for protein synthesis, protein folding, lipid synthesis and intracellular calcium homeostasis [9,10]. 

The abnormality of calcium balance or protein folding in ER may result in ER stress and this subsequently induces self-rescuing or destructive responses in cells. Processes of mitochondrial dysregulation have been demonstrated as major events during apoptosis while the Bcl-2 family, like Bax and Bak, is related to the alteration of mitochondrial membrane potential and the release of mitochondrial apoptotic factors [11]. Cytochrome *c* freed from the mitochondrial inter-membrane spaces initiates the serial reactions leading to apoptosis. The serial reactions begin with the activation of caspase-9, and further activation of the downstream effector caspase-3; activated caspase-3 then cleaves and activates poly ADP-ribose polymerase (PARP) [11,12].

Recently, many studies have analyzed active compounds from soft coral seeking new therapeutic drug candidates for the inhibition or prevention of cancer development [13,14,15,16]. Sinulariolide, an active compound isolated from the cultured soft coral *Sinularia flexibilis* [17] has various biological properties, including anti-microbial and anti-cancer activities, particularly in bladder cancer and melanoma [18,19]. In the current research, the effects of sinulariolide on hepatoma cells were evaluated through cell viability, colony formation, flow cytometry, TUNEL/DAPI stain and Annexin V/PI stain analysis. The results indicated that sinulariolide has the apoptosis-inducing ability and the anti-tumor properties on HA22T cells. The potential pathways of apoptosis stimulated by sinulariolide in HCC cells were determined by mitochondrial membrane potential measurement, apoptotic inhibition test and immunoblotting. Crucial data was obtained to reveal the cytotoxic activities and several apoptotic pathways of sinulariolide in HCC cells *in vitro*. Overall, these results could provide valuable information for drug development or potential strategies against human HCC.

## 2. Results and Discussion

### 2.1. The Cytotoxic Effects of Sinulariolide on Different Hepatocellular Carcinoma Cell Lines

**Figure 1:**
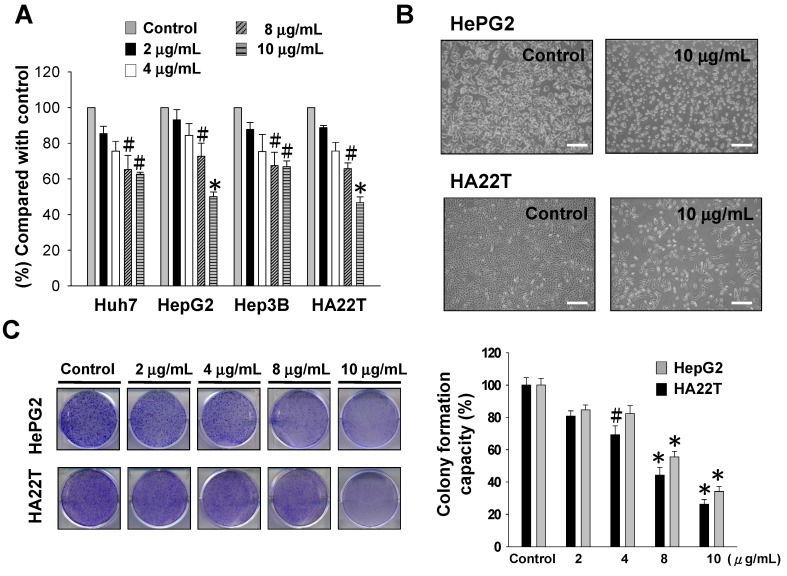
Evaluation of the cell cytotoxicity and colony formation effects of sinulariolide on hepatocellular carcinoma cell lines. (**A**) The viable cell number of different HCC cell lines was dose-dependently suppressed by MTT assay. The results shown are representative of three independent experiments (^#^ *p* < 0.05, * *p* < 0.001 compared with the control). HA22T and HepG2 cell lines were more sensitive to sinulariolide treatment. (**B**) The morphological change of HA22T and HepG2 cells upon sinulariolide treatment. Scale bar = 20 μm (**C**) Cells were seeded in six-well plates. After 24 h of incubation, cells were treated with serial concentrations of sinulariolide. The number of colony formation was counted as described in the Experimental section. The results shown are representative of three independent experiments (^#^ *p* < 0.05, * *p* < 0.001 compared with the control).

To explore the potential cytotoxic effect of sinulariolide on the HCC cells, MTT assay and colony formation assay were performed. The cytotoxic effect of sinulariolide on four HCC cell lines (HepG2, Hep3B, HA22T and Huh7 cells) were performed in this study. HCC cells were treated with various concentrations (2, 4, 8, 10 μg/mL) of sinulariolide for 24 h. Sinulariolide treatment clearly reduced cell viability in all HCC cell lines in a dose-dependent manner. The result showed that HA22T and HepG2 cells were more sensitive in response to the treatment of sinulariolide (Figure 1A). The IC_50_ values of sinulariolide treatment in four HCC cell lines are shown in Table 1.

**Table 1:** IC50 values of sinulariolide treatment in four HCC cell lines.

Cell line	IC_50_ (μg/mL)
Huh7	12.31 ± 0.25
HepG2	10.34 ± 0.34
Hep3B	16.52 ± 0.13
HA22T	8.46 ± 0.05

The cell morphology was investigated and compared between control and sinulariolide-treated HA22T and HepG2 cells using the inverted light microscopy. Microscopic observations revealed that HA22T and HepG2 cell population decreased after 10 μg/mL sinulariolide treatment (Figure 1B). In HA22T and HepG2 cells, sinulariolide showed dose-dependent inhibitory effects on the colony formation (Figure 1C). In HA22T cells, the decreased numbers of colonies formed at sinulariolide concentrations of 2, 4, 8 and 10 μg/mL were 20, 31, 56 and 74% respectively. In HepG2 cells, the decreased numbers of colonies formed at sinulariolide concentration of 2, 4, 8 and 10 μg/mL were 16, 18, 45 and 66% respectively. Comparing the HA22T and HepG2 cell lines, HA22T cells were more sensitive to sinulariolide treatment.

### 2.2. Sinulariolide Induced Apoptosis of HA22T Cells

**Figure 2:**
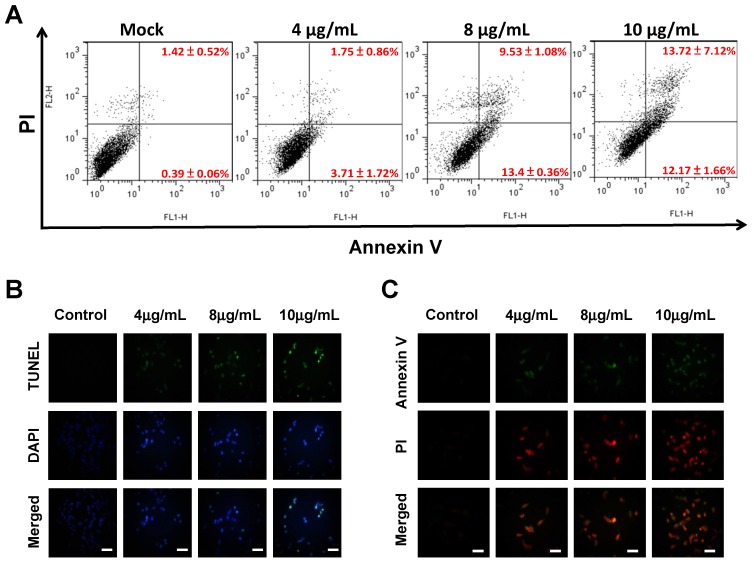
Sinulariolide-induced apoptosis of HA22T cells. (**A**) Detection of apoptotic HA22T cells after sinulariolide treatment (0, 4, 8 and 10 μg/mL) by Annexin V- fluoresceinisothiocyanate (FITC)/porpidium iodide (PI) analysis. Sinulariolide induced early and late apoptosis in a dose-dependent manner. Bottom right quadrants, early apoptotic cells; top right quadrants, late apoptotic cells. (**B**) AnnexinV-FITC/PI analyses of apoptotic HA22T cells upon sinulariolide treatment. The HA22T cells were stained by PI (red) and Annexin-V (green) after different concentrations of sinulariolide treatment. (**C**) Detection of apoptotic cells by TUNEL and DAPI staining assay. HA22T cells were treated with sinulariolide at the final concentration of 4, 8 and 10 μg/mL for 24 h. The cells were harvested for TUNEL and DAPI staining as described in the Experimental section. Scale bar = 50 μm.

To investigate whether sinulariolide induced HA22T cell apoptosis, HA22T cells were treated with sinulariolide and stained with flow cytometry based-annexin V-FITC/PI double staining, and analyzed with flow cytometer. After treatment with 0, 4, 8 and 10 μg/mL of sinulariolide, the presences of early apoptosis/later apoptosis were 0.39%/1.42%, 3.71%/1.75%, 13.4%/9.53% and 12.1%/13.7%, respectively (Figure 2A). These data showed that sinulariolide efficiently induced apoptosis of HA22T cells. To further validate apoptotic effect of sinulariolide on the HA22T cells, annexin V-FITC/PI double staining, Terminal deoxynucleotidyltransferase UTP nick end labeling (TUNEL) and 4’,6-diamidino-2-phenyl iodide (DAPI) stained assays were performed. Some massive apoptotic bodies were observed in HA22T cells treated with 10 μg/mL of sinulariolide (Figure 2B,C). Altogether, these results demonstrated that sinulariolide induced both early and late apoptosis in HA22T cells, and the apoptotic effect was dose-dependent.

**Figure 3:**
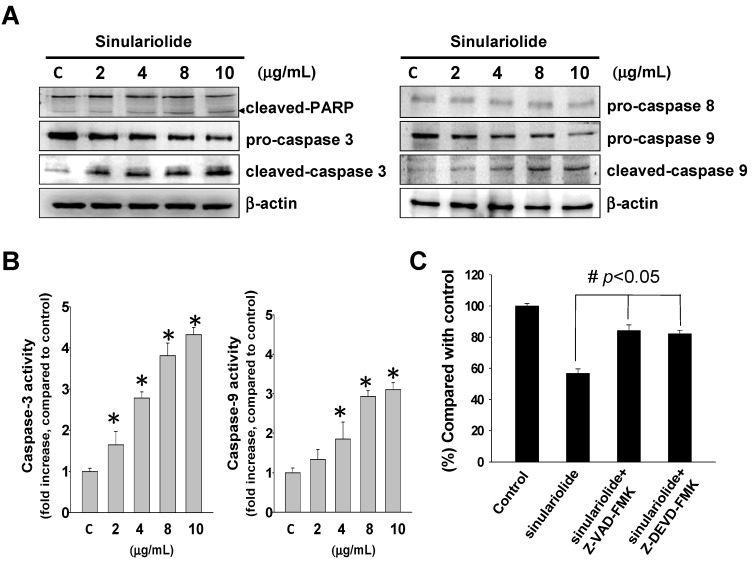
Sinulariolide induced apoptosis through activated caspase cascade pathway. HA22T cells were treated with sinulariolide (0, 2, 4, 8 and 10 μg/mL) for 24 h. (**A**) The detection of mitochondrial related apoptosis proteins were performed by western blotting analysis using specific antibodies as indicated. The results showed the changes of caspase-3, caspase-8, caspase-9, cleaved-caspase-3 and cleaved-caspase-9 expression in HA22T treated with sinulariolide. β-actin was used as the protein loading control. (**B**) Caspase-3 and caspase-9 activities were increased after sinulariolide treatment. The results shown are representative of three independent experiments (^#^ *p* < 0.05, * *p* < 0.001 compared with the control). (**C**) HA22T cells were pre-treated with two cell-permeant pan caspase inhibitors, Z-VAD-FMK and Z-DEVD-FMK, before sinulariolide treatment. The cells were then harvested at 24 h and further MTT assay was performed for the evaluation of cell viability. The related cell viabilities were determined from three independent experiments (^#^ *p* < 0.05, compared with the control).

We further investigated the effect of sinulariolide on the activation of caspase and PARP cleavage. We examined the PARP, pro-caspase-3, cleaved-caspase-3, pro-caspase-9, cleaved-caspase-9 and pro-caspase-8 by western blotting assay after sinulariolide treatment. In Figure 3A, western blotting result showed decreased expression levels of pro-caspase-3, pro-caspase-8 and pro-caspase-9 in the sinulariolide treated cells. The increased expression level of cleaved-caspase-3, cleaved-caspased-9 and cleaved-PARP (89 kDa proteolytic fragments) after sinulariolide treatment were observed. These results showed that sinulariolide could activate caspase-dependent pathway. Caspase-8 was not changed after sinulariolide treatment (Figure 3A). Sinulariolide also induced caspase-3 and caspase-9 activity (Figure 3B).

To determine whether sinulariolide induced cell apoptosis through caspase activation, cells were pre-treated with Z-DEVD-FMK (caspase-3 inhibitor) and Z-LEHD-FMK (caspase-9 inhibitor) before sinulariolide treatment. The result showed that pre-treatment with Z-DEVD-FMK and Z-LEHD-FMK significantly inhibited the sinulariolide-induced cell apoptosis (Figure 3C). These data suggested that caspase-3 and caspase-9 were involved in the sinulariolide-induced cell apoptosis in HCC cells.

### 2.3. Treatment of Sinulariolide Causes the Mitochondrial Depolarization and Activated the Mitochondrial-Related Apoptotic Pathway in HA22T Cells

**Figure 4:**
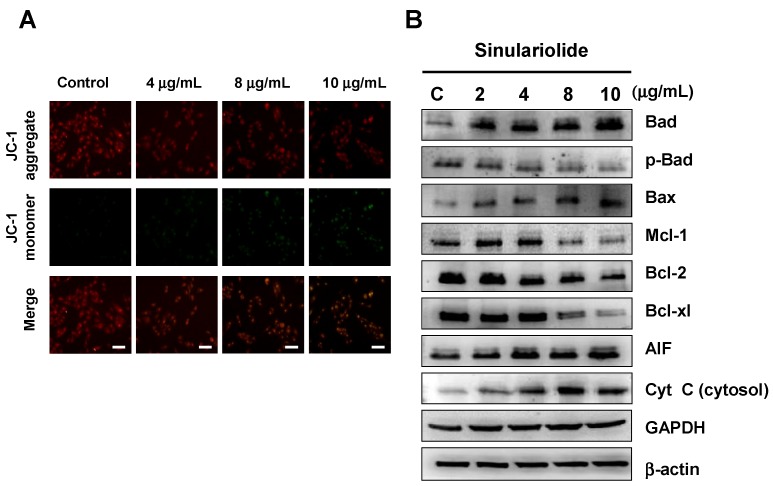
Sinulariolide induced mitochondrial membrane depolarization in the HA22T cells. (**A**) Cells were treated as indicated, stained with JC-1 dye, incubated with cells for 20 min at 37 °C, 5% CO_2_ and imaged under fluorescence microscope at the emission wavelength of 580 nm (red, upper panels) and 530 nm (green, lower panels). Scale bars = 50 μm. (**B**) Western blotting data showed the changes of Bax, Bad, p-Bad, Bcl-2, Bcl-xL, Mcl-1, cytosolic cytochrome *c*, and AIF expression in HA22T cells treated with different concentrations of sinulariolide. β-actin was used as the cytosol marker internal control. GAPDH was used as the mitochondrial marker internal control.

The involvement of changes in mitochondrial membrane potential (ΔΨm) in the role of mitochondrial-related apoptosis initiation has received growing attention. In this study, we measured the change in mitochondrial membrane potential (ΔΨm) induced by sinulariolide with JC-1 dye. In the sinulariolide-treated HA22T cells, there were significantly increased green fluorescence signals while reduced red fluorescence signals by fluorescence microscopy, suggesting the loss of ΔΨm with sinulariolide treatment (Figure 4A).

The involvements of caspase-3 and caspase-9 were demonstrated in the previous section, and these suggested that mitochondrial-related apoptotic pathway plays a critical role in the sinulariolide-induced cell apoptosis. Several mitochondrial-related apoptotic markers were analyzed to verify the assumption, including Bcl-2, Bcl-xL, Mcl-1, Bax, Bad, p-Bad, cytosolic cytochrome *c* and AIF. The results indicated that sinulariolide induced the cell apoptosis through the mitochondrial-related apoptotic pathway (Figure 4B).

### 2.4. Treatment of Sinulariolide Induces the Activation of ER Stress Pathway in HA22T Cells

**Figure 5:**
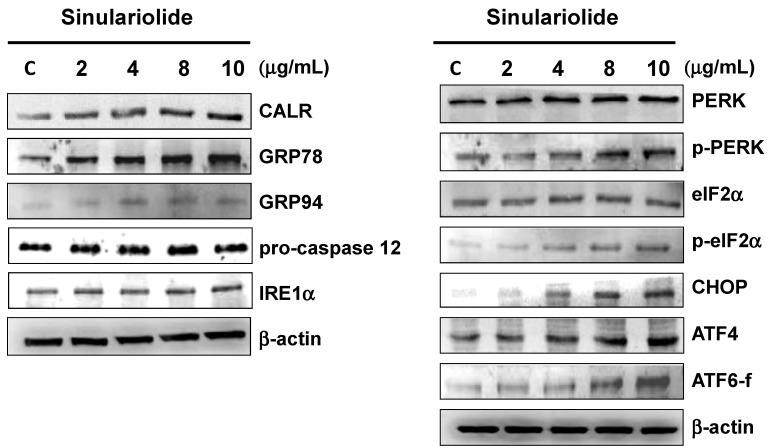
Exposure to sinulariolide stimulated the factors of ER stress-mediated apoptotic pathway. The changes of ER stress related chaperones including CALR, GRP78 and GRP94 and ER stress sensors IRE1α, caspase-12, PERK, p-PERK, e-IF2α, p-eIF2α, ATF4, ATF6 fragment and CHOP were represented by immunoblotting. β-actin was used as the internal control.

In the current study, regulations of three ER sensors, IRE1α, PERK, and ATF6 as well as the caspase-12, GRP78, GRP94 and CALR were verified by western blot. The expression of ER chaperones GRP78, GRP94, and CALR after treating sinulariolide was dose-dependently up-regulated. Caspase-12 and IRE1α were not changed after sinulariolide treatment. In addition, the expression levels of p-PERK and p-eIF2α were up-regulated, but PERK and elF2α levels remained unchanged normal forms. The transcription factor ATF4, a downstream signal of PERK-eIF2α pathway, was increased after sinulariolide treatment in HA22T cells. The expression of CHOP, a nuclear protein induced by ER stress, was also increased (Figure 5).

**Figure 6:**
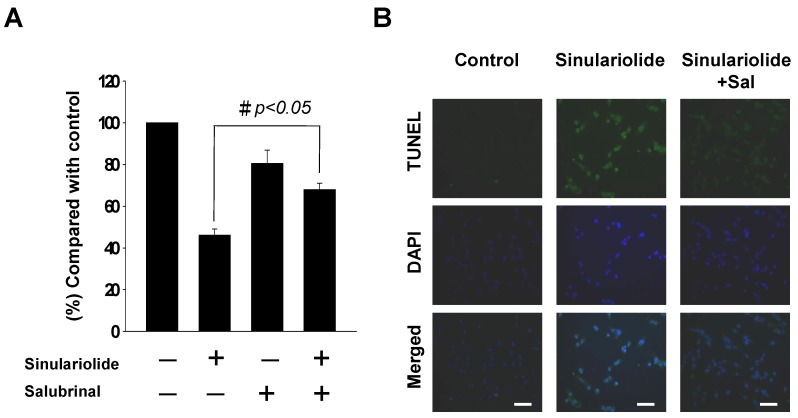
Sinulariolide induced HA22T cell apoptosis through ER stress pathways. (**A**) Salubrinal increased cell viability of the sinulariolide-treated HA22T cells. The results shown are representative of three independent experiments (^#^ *p* < 0.05, compared with the control). (**B**) The TUNEL and DAPI staining showed a decrease in sinulariolide-induced apoptotic cascade with ER stress inhibitor, salubrinal.

We further verified the activation of ER stress-mediated apoptotic pathway by performing the ER stress inhibitory test. The HA22T cells were pre-treated with salubrinal, an ER stress inhibitor targeting eIF2α dephosphorylation, before sinulariolide treatment. With 10 μM salubrinal pre-treatment, the cell viability increased from 44% to 68% in the sinulariolide-treated HA22T cells (Figure 6A). Further, TUNEL and DAPI assays were performed to detect DNA fragmentation after sinulariolide-induced apoptosis. The positively TUNEL-stained HA22T cells after sinulariolide treatment was shown in Figure 6B. After pre-treatment with salubrinal, the number of positively TUNEL-stained HA22T cells was significantly decreased. This suggested that ER stress was involved in sinulariolide-induced cell apoptosis.

### 2.5. Discussion

The current study demonstrated that sinulariolide has cytotoxic effects and induces apoptosis of the HCC cells. Sinulariolide treatment clearly reduced cell viability on four HCC cell lines (HepG2, Hep3B, HA22T and Huh7 cells) in a dose-dependent manner by MTT assay (Figure 1A). Compared with MTT and colony formation assays, HA22T cells were more sensitive to sinulariolide treatment. Treatment with sinulariolide induced HA22T cell apoptosis, as demonstrated by flow cytometry, TUNEL/DAPI stain and Annexin V/PI stain analyses. The flow cytometric results indicated that sinulariolide induced both early and late apoptosis (Figure 2A).

Induction of apoptosis is directed by intrinsic or extrinsic pathways [6,7,20]. Mitochondria play an important role in the initiation of intrinsic pathway with stress. Recent studies suggested endoplasmic reticulum (ER) could also initiate apoptotic cascade in response to stress [20,21]. In response to stress, pro-apoptotic proteins Bid, Bax and Bak are activated, while anti-apoptotic proteins like Bcl-xL, Bcl-2 and Mcl-1 are suppressed. These changes in apoptotic-related proteins and increased ratio of Bax/Bcl-2 result in alteration of mitochondrial membrane potentials, and cytochrome *c* and apoptosis-inducing factor (AIF) are released from the mitochondrial inter-membrane spaces into cytosol [11]. The release of cytochrome *c* from mitochondrial inter-membrane spaces initiates further caspase pathway by binding to Apaf1 and caspase-9, forming apoptosome, and leads to the activation of downstream effector caspase-3 [22,23]. The activated caspase-3 then inactivated poly-(ADP-ribose) polymerase (PARP) through caspase cleavage [11]. In this study, sinulariolide suppressed anti-apoptotic factors Bcl-2, Bcl-xL and Mcl-1, and promoted pro-apoptotic factors Bad and Bax (Figure 4). The change in mitochondrial membrane potential was also observed after sinulariolide treatment, leading to cytochrome *c* and AIF release. The levels of activated caspase-3 and caspase-9, and cleaved PARP were also increased in a sinulariolide concentration-dependent manner (Figure 3). The present data supported that sinulariolide can induce apoptosis of HA22T cells through the activation of mitochondria-related apoptotic pathway.

The ER is a central organelle responsible for regulating intracellular Ca^2^^+^ homeostasis, protein folding, protein synthesis and lipid synthesis [10,24]. The ER is known to respond to various stressors and slows the protein folding process. In the present study, ER stress is also observed to involve in the apoptotic events after sinulariolide treatment in HCC cells [8,24]. The activation of UPR critically requires glucose-regulated protein 78 (GRP78) to regulate three ER-resident transmembrane sensors, inositol-requiring enzyme 1 alpha (IRE1α), RNA-dependent protein kinase (PKR)-like ER kinase (PERK), and activating transcription factor 6 (ATF6). Under ER stress, these sensors trigger responses toward restoration of normal ER function or apoptosis [25]. Among these, IRE1α is the most conserved UPR [26]. 

To verify the involvement of ER stress in the sinulariolide-induced apoptosis, we further analyzed UPR sensors, including activating transcription factor 4 (ATF4), a downstream effector of the PERK-eIF2α signal pathway. ATF4 translocates into nucleus, leading to further downstream CHOP gene expression. CHOP is identified to be one of the proteins that mediate ER stress-induced apoptosis [27,28]. In the present study, the expression of ATF4 and CHOP were significantly increased after sinulariolide treatment (Figure 5), indicating the role of ATF4 in the regulation of CHOP expression through PERK-eIF2α signal pathway following treatment of sinulariolide.

We also found an increased expression of ATF6, another UPR sensor, after treating sinulariolide (Figure 4A). ATF6 is another UPR sensor that is induced through ER stress. Activated ATF6 is a transcription factor that regulates ER chaperones, X box-binding protein 1 (XBP1) [29] and CHOP expression [30,31]. In the present study, we also found increased expression of fragmented ATF6 by immunoblotting (Figure 5), indicating the role of ATF6 in the regulation of CHOP expression in response to ER stress induced by sinulariolide treatment.

To confirm the involvement of PERK-eIF2α signal pathway in the ATF4 regulated CHOP expression, we pre-treated hepatoma cells with an ER stress inhibitor, salubrinal, that targeted specific phosphatase of eIF2α, blocking the dephosphorylation of p-eIF2α [32]. The result showed that the degree of cell death induced by sinulariolide was partially reversed by salubrinal pre-treatment (Figure 6). In our current study, we found that PERK/eIF2α/ATF4/CHOP signal pathway was not the only pathway involved in the sinulariolide induced cell apoptosis. The ATF6/CHOP pathway was also involved. Thus, inhibition of p-eIF2α dephysphorylation by salubrinal could only partially abrogate sinulariolide induced cell apoptosis by around 24%. However, the expressions of IRE1α and caspase-12 were not changed following sinulariolide treatment.

In conclusion, it is suggested that sinulariolide is capable of inducing HCC cell apoptosis via both mitochondrial-related apoptotic pathway and ER-stress induced apoptotic pathway. Further study is indicated to determine the cytotoxic effect of sinulariolide to hepatoma *in vivo*. 

## 3. Experimental

### 3.1. Materials

Rabbit anti-human poly ADP-ribose polymerase-1 (PARP-1), eIF2-α, and glucose-related protein 78 (GRP78), glucose-related proteins 94 (GRP94) antibodies were obtained from Epitomics (Burlingame, CA, USA). Rabbit anti-human cleaved-ATF6 and ATF4 antibodies were obtained from ProteinTech Group (Chicago, IL, USA). Rabbit anti-human pro-casapse-3, pro-caspase-8, pro-caspase-9, cleaved caspase-3, cleaved caspase-9, Bax, Bcl-xL, Bcl-2, Bad, p-Bad, PERK, p-PERK, p-eIF2-α, calreticulin (CALR) cytochrome *c* and GAPDH antibodies were obtained from Cell Signaling Technology (Danvers, MA, USA). Dimethyl sulfoxide (DMSO), 3-(4,5-Dimethylthiazol-2-yl)-2,5-diphenyltetrazolium bromide (MTT), Protease inhibitor cocktail, salubrinal (eIF2-α inhibitor) and rabbit anti-human β-actin antibodies were obtained from Sigma (St. Louis, MO, USA). PVDF (polyvinylidenedifluoride) membranes and goat anti-rabbit and horseradish peroxidase conjugated IgG were obtained from Millipore (Bellerica, MA, USA). Mitochondria/cytosol fractionation kit was obtained from BioSource International (Camarillo, CA, USA). Chemiliminescent HRP substrate was purchased from Pierce (Rockford, IL, USA). Annexin V–FITC Apoptosis Detection kit was obtained from Pharmingen (San Diego, CA, USA). JC-1 (5,5,6,6-tetrachloro-1,1,3,3-tetraethylbenzimidazolcarbocyanine iodide) fluorescent kit was obtained from Biotium (Hayward, CA, USA).DAPI fluorescent kit and TUNEL fluorescent kit were obtained from Promega (Madison, WI, USA). Sinulariolide was extracted from cultured soft coral *Sinularia* *flexibilis*, following the protocol described by Su *et al*. [17], and dissolved in DMSO*.*

### 3.2. Cell Culture and MTT Assay

HepG2, HepG3, Huh7 and HA22T HCC cell lines were purchased from Food Industry Research and Development Institute (Hsinchu, Taiwan) and were cultured in DMEM (Biowest, Nuaillé, France) containing 4 mM L-glutamine, 1 mM sodium pyruvate, 100 μg/mL streptomycin, 100 U/mL penicillin and 10% (v/v) fetal bovine serum, in a 37 °C humidified atmosphere with 5% CO_2_. Four HCC cell lines were used to determine the cytotoxic effect of sinulariolide on HCC cells. Each cell line was treated with various concentrations of sinulariolide (2, 4, 8, 10 μg/mL) and harvested after incubation for 24 h. The cell viability of HCC cells after sinulariolide treatment was examined by colorimetric MTT assay, as described in our previous study [33]. HCC cells were seeded on 24-well culture plates at a density of 1 × 10^5^ cells/well. After the addition of 2–10 μg/mL sinulariolide for 24 h, 50 μL of MTT solution (1 mg/mL in PBS) was added to each well. Cells treated with DMSO without sinulariolide were used as blank control. The plates were then incubated at 37 °C for 4 h. Then cells were lysed with 200 μL DMSO. The optimal density (O.D) was measured at 595 nm by a microtiter ELISA reader (Bio-Rad, Hercules, CA) with DMSO as the blank. All the experiments were repeated three times.

### 3.3. Colony Formation Assay

Colony formation assay was performed following the previous study [34]. HA22T (2000 cells/well) and HepG2 (2000 cells/well) cells were seeded in 24-well plates. After 24 h, cells were treated with various concentrations (2, 4, 8 and 10 μg/mL final concentration) of sinulariolide in 2 mL of serum complete media. After culture for 10 days, media were removed and colonies were washed with PBS. The colonies were fixed with methanol for 15 min and stained with 0.15% crystal violet for 10 min. The colonies were counted and scanned with a high-resolution scanner Scan Maker 9800XL (MiCROTEK, Hsinchu, Taiwan). The cells observed with no connection in between is calculated as one colony. Regarding the structure formed by the connection of cells, we estimated the shape and the size of it to figure out its cell count (for overlapping ones we calculate them as one cell). 

### 3.4. Quantitative Detection of Apoptosis by Flow Cytometry

To determine the apoptosis induced by sinulariolide in HA22T cells, the annexin V–FITC Apoptosis Detection kit was used following the protocol described in the previous study [33]. A total of 1 × 10^6^ cells were seeded onto 5 cm petri-dish and treated with various concentrations of sinulariolide or 0.1% DMSO for 24 h, and cells were subsequently collected and fixed in 70% cold ethanol at 4 °C overnight. After washing, the cells were stained with annexin V-FITC and propidium iodide (PI) for 30 min at 37 °C following the protocol provided by the manufacturer. FACScan flow cytometer and Cell-Quest software (Becton-Dickinson, Mansfield, MA, USA) were used to analyze the apoptotic cells. 

### 3.5. TUNEL Assay and DAPI Staining

HA22T cells (1 × 10^5^ cells/well) cultured in 12-well plates were treated with 4, 8, and 10 μg/mL sinulariolide for 24 h, while adding DMSO for the control. Cells in each concentration and control were fixed with ice-cold 4% paraformaldehyde in PBS solution for 15 min, washed with PBS and then stained with 2 μg/mL DAPI for 20 min at 37 °C. TUNEL was performed according to the manufacturer’s manual for DeadEnd™ Fluorometric TUNEL System (Promega). The cells were photographed under a fluorescence microscopy (Olympus IX71 CTS, Chinetek Scientific, Hong Kong, China).

### 3.6. JC-1 Staining

Following treatment with different concentrations of sinulariolide, the changes in the mitochondrial membrane potential (ΔΨm) were assessed, using cationic dye JC-1 for staining. In cells with healthy mitochondria, JC-1 accumulated in mitochondria, indicating red fluorescence emission (560 nm). As mitochondrial membrane potentials changed, JC-1 uptake was distributed in cytoplasm, indicating green fluorescence emission (530 nm). HA22T cells were pretreated with sinulariolide, and incubated with 10 mg/mL JC-1 at 37 °C in the dark for 30 min. Cells were washed twice with serum-free medium and observed under fluorescence microscopy (Olympus IX71 CTS).

### 3.7. Caspase-3 and Caspase-9 Activity Assay

Caspase-3 and caspase-9 fluorescence assay kits were purchased from Cell Signaling Technology. For caspase-3 activity measurement, cells were collected and resuspended in lysis buffer on ice for 30 minutes, followed by centrifugation at 12,000 rpm at 4 °C to obtain supernatant. Reaction mixture and caspase-3 substrate (Ac-DEVD-AMC) were added into cell supernatant and incubated at 37 °C for 2 h. The caspase-3 activity was measured by a fluorometer (Turner Biosystem, Promega), with fluorescence excitation wavelength of 350 nm and emission wavelength of 420 nm. For caspase-9 activity measurement, cells were resuspended in lysis buffer on ice for 30 min, followed by centrifugation at 12,000 rpm at 4 °C to obtain supernatant. Reaction mixture and caspase-9 substrate (Ac-LEHD-AFC) were added into cell supernatant and incubated at 37 °C for 2 h. The caspase-9 activity was measured by fluorometer (Turner Biosystem), with fluorescence excitation wavelength of 400 nm and emission wavelength of 505 nm. 

### 3.8. Mitochondria and Cytosol Fractionation

The mitochondria and cytosol fractions were separated with commercial mitochondria/cytosol fractionation kit (BioSource International, Camarillo, CA, USA), following the methods previously reported [19].

### 3.9. Western Blot Analysis

Western blot analysis was performed following the methods previously reported [35]. The treated samples and the control samples (25 μg) were separated by 12.5% SDS-PAGE, and then transferred onto PVDF membrane for 1.5 h at 400 mA using Transphor TE 62 (Hoeffer) and then proteins transfer checked by staining with Ponceau S solution. The membranes were incubated with 5% dehydrated skim milk to block nonspecific protein bindings, and then incubated with primary antibodies at 4 °C overnight. The primary anti-human PARP-1, pro-caspase-3, cleaved-caspase-3, pro-caspase-9, cleaved-caspase-9, pro-caspase-8, pro-caspase-12, cytochorme *c*, Bax, Bad, p-Bad, Bcl-2, Bxl-xL, Mcl-1, AIF, CALR, eIF2α, p- eIF2α, IRE1α, PERK, p-PERK, CHOP, ATF4, cleaved-ATF6 and β-actin antibodies were used. The second antibodies (horseradish peroxidase conjugate goat anti-rabbit, 1:5,000 in blocking solution) were added and incubated for 2 h at 4 °C and then visualized using chemiluminesence (Pierce). The western blot data were quantified using Image J software.

### 3.10. Statistical Analysis

Data of MTT assay, colony formation and flow cytometric analysis were pooled from three independent experiments. The results were expressed as mean ± standard error of mean (SEM). Data acquisition and analysis of variance (ANOVA) was carried out by the Tukey-Kramer test, using GraphPadInStat 3 software (San Diego, CA, USA) [16].

## 4. Conclusions

**Figure 7:**
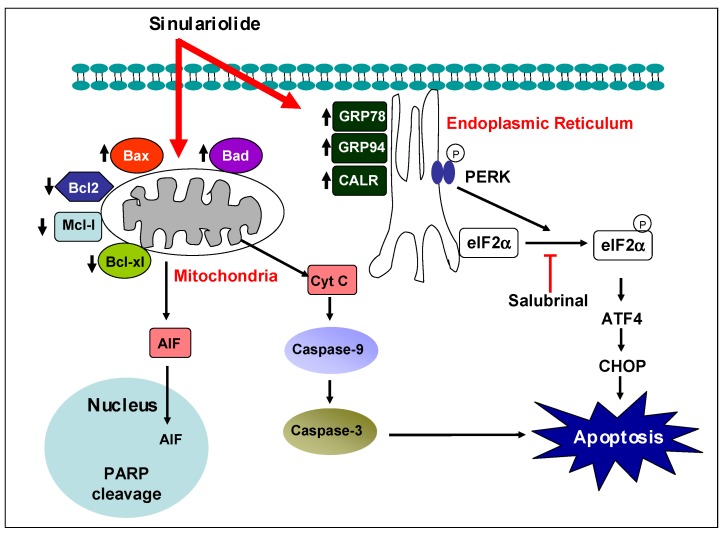
Illustration of how sinulariolide induces cellular apoptosis through mitochondrial-related apoptosis and PERK/eIF2α/ATF4/CHOP pathway on HA22T cells.

In conclusion, our results demonstrated that sinulariolide may have potential selective cytotoxic effects towards HA22T cells by mediation through the induction of cell apoptosis. Mitochondrial-related apoptosis and PERK/eIF2α/ATF4/CHOP signal pathway were induced by sinulariolide (Figure 7). The findings in our current study also benefit the HCC pharmaceutical development in the clinical setting. Hence, further evaluation of the anti-HCC effect of sinulariolide is needed to determine the *in vivo* efficacy and pharmacokinetic effects of this naturally extracted compound.
